# Supplementary material for: Disparities in the prevalence of screened depression at different altitudes in Peru: A retrospective analysis of the ENDES 2019
Source: PLoS One. 2022 Dec 21;17(12):e0278947. doi: 10.1371/journal.pone.0278947 (PMC9770421; doi:10.1371/journal.pone.0278947)
Supplement: Attachment — Submitted filename: Response to Reviewers.docx [file pone.0278947.s005.docx]

**Response to Journal Requirements**

1. Please ensure that your manuscript meets PLOS ONE's style requirements, including those for file naming.

**Response:** *We have ensured our manuscript meets the journal’s requirements.*

2. Please clarify the sources of funding (financial or material support) for your study. List the grants or organizations that supported your study, including funding received from your institution.

**Response:** *We have clarified the sources of funding in the cover letter (“The authors received no specific funding for this work”).*

3. State what role the funders took in the study. If the funders had no role in your study, please state: “The funders had no role in study design, data collection and analysis, decision to publish, or preparation of the manuscript.”

**Response:** *The study has received no funding.*

4. If any authors received a salary from any of your funders, please state which authors and which funders.

**Response:** *No author received a salary from any funder.*

5. If you did not receive any funding for this study, please state: “The authors received no specific funding for this work.”

**Response:** *The authors received no specific funding for this work.*

**Response to Reviewers**

**Reviewer 1**

6. The authors may a bit more in detail discuss (in the intro section) why high-altitude living (and hypobaric hypoxia) may provoke depression. Based on that it should be possible to state a clear hypothesis. You may consider (beside others) the following reviews: PMID: 29517615, PMID: 35661753).

**Response:** *Thank you for your feedback. We have better explained the mechanism of hypobaric hypoxia in the development of depression, including the suggested reviews (lines 54-61).*

7. The procedure of recruitment (and inclusion) of study participants may be better explained (possibly including a figure), e.g., total persons eligible, inclusion, exclusion criteria, number of respondents, etc. Are you sure that potential inter-correlations between predictors with have been sufficiently considered by your statistical approach?

**Response:** *Thank you. We have better explained the recruitment and inclusion of study participants (lines 85-123 and Figure 1). In addition, we have assessed the Variance Inflation Factor (VIF) and other collinearity diagnostic tests, showing no intercorrelations between predictors (lines 215-217).*

8. The increase in depressive symptoms is not much different from 1500-2500 m to > 2500 m. If hypoxia would represent a major explaining mechanism, one would expect an increase of symptoms with altitude.

**Response:** *Thank you. We have better discussed the role of other factors affecting our results (lines 271-284).*

9. There are several other environmental factors that could influence depression, e.g., UV-radiation, temperature, etc., but also life-style factors (like diet, physical activity, alcohol consumption, etc.) and employment status. You may elaborate a bit on that.

**Response:** *Thank you. We have discussed the role of other environmental and life-style factors (lines 253-284).*

10. In my opinion, your data do not support the conclusion that the higher the altitude the more frequent are depressive symptoms. Please, check and explain.

**Response:** *Thank you. We have revised the conclusion as suggested.*

11. A more clinically useful approach should be provided how to implement your findings.

**Response:** *Thank you. We have provided information on how these findings could be implemented in the clinical context (lines 338-343).*

12. Please, do not forget to mention other (here not or less considered) risk factors like social support, sleep quality/duration, physical activity…

**Response:** *Thank you. We have mentioned other important risk factors as suggested (lines 253-284).*

**Reviewer 2**

13. The title is very unspecific and could be more "interesting" to the reader, for example, I suggest ideas: Analysis of the differences in the depression score (PHQ-9) at different altitudes in Peru" or something like that.

**Response:** *Thank you for your feedback. We have modified the title as suggested.*

14. The STROBE reporting format is incomplete, there is missing information and I suggest reviewing step by step the available template.

**Response:** *Thank you. We have completed the information using the STROBE reporting guideline.*

15. Third, it seems to me that a work like this could benefit from a figure showing the differences (pictorially) between the different elevations.

**Response:** *Thank you. We have added a figure (Figure 2) showing the differences in the prevalence of depressive symptoms according to the altitude.*

16. The justification in relation to elevation due to lower PO2 would seem to be very theoretical, there is evidence to suggest that elevation is associated with such findings, among them:

About PO2 and environment:

<https://www.ncbi.nlm.nih.gov/pmc/articles/PMC6420699/>

About suicide at high altitude:

<https://pubmed.ncbi.nlm.nih.gov/29017474/>

About altitude, depression and self-perception:

<https://pubmed.ncbi.nlm.nih.gov/35020475/>

**Response:** *Thank you. We have discussed the evidence on altitude and depression (235-284). We have also added the suggested references in the discussion (lines 251-253 and 286-294).*

17. Was the questionnaire conducted in all of Peru? How were the locations classified ? By city altitude ? By canton? By parishes?

**Response:** *Thank you. The questionnaire was conducted in all the regions of Peru. We have clarified/added the requested information in lines 85-110.*

18. On the other hand, did they use any standard classification about altitude and its ranges? in general there are several classifications (<2,500 m low vs high > 2,500) or the category of low, moderate, high and very high (read this article <https://www.frontiersin.org/articles/10.3389/fphys.2021.733928/full>)

**Response:** *Thank you. We adapted the standard classification on altitude based on the article of Barry & Pollard, as explained in lines 139-141.*

19. It is noteworthy that there is less depression above 2500 m and that moderate altitudes have more, so it is essential to categorize the variables on the altitude properly.

**Response:** *Thank you. We have followed the classification of previous literature for the cutoff value of 2500 m (as explained in Comment #18). In addition, we believe this similarity was subtle and the confidence interval is not so precise to interpret an increasing pattern of depression by altitude. The possible reasons for this finding are explained in lines 257-284.*
