# Supplementary material for: Disparities in the prevalence of screened depression at different altitudes in Peru: A retrospective analysis of the ENDES 2019
Source: PLoS One. 2022 Dec 21;17(12):e0278947. doi: 10.1371/journal.pone.0278947 (PMC9770421; doi:10.1371/journal.pone.0278947)
Supplement: Attachment — Submitted filename: Response to Reviewers.docx [file pone.0278947.s006.docx]

**Response to reviewer 2**

1. Your paper is not in the correct publication format for this type of study, and you should review the STROBE guidelines and follow each criterion to submit your paper.

***Response: Thank you. We have followed the STROBE guideline as suggested. We welcome any additional details that may be necessary.***

2. Second, their work of revision of the literature is inferior; they have not delved into the subject of the physiopathology of the possible causes behind depression in the inhabitants of high altitudes, nothing is said about the metabolism of serotonin at high altitudes, the role of neurodevelopment is not mentioned, there is no mention of self-perception at high altitude, suicide rates at high altitudes use US data (which has almost no populations living at high altitude) and so on (refer to the literature for further information <https://pubmed.ncbi.nlm.nih.gov/35020475/>

***Response: Thank you. This information was stated in line 54. However, we have added more detail in lines 71 and 76.***

3. Third, the altitude ranges are arbitrary; why do they use that range? Where do they get it from? The altitude ranges have been described previously and are well known among those who do high altitude medicine, they can use the simple range of low and high altitude (< or > 2,500 m) or the range of low (<1500), moderate (1500-2500), high altitude (2500-3500) and very high altitude (3,500 to 4300 m) see the following work for further detail <https://pubmed.ncbi.nlm.nih.gov/34675818/>

***Response: Thank you for your feedback. We understand your point, but we disagree that the altitude ranges used in the study are arbitrary, as it follows standard classifications (shown in cite 14). Additionally, they were considered in a previous Peruvian report (cite 15) and other articles used similar categorizations (see*** [***https://www.ncbi.nlm.nih.gov/pmc/articles/PMC8330906/***](https://www.ncbi.nlm.nih.gov/pmc/articles/PMC8330906/) ***and*** [***https://www.ncbi.nlm.nih.gov/pmc/articles/PMC8647184/***](https://www.ncbi.nlm.nih.gov/pmc/articles/PMC8647184/)***). Therefore, we feel it is of value for the literature. Despite this, we have considered the suggested cut-off points and presented two additional analyses with both simple and extended altitude ranges (Please see Line 149, Line 230, and Supplementary Tables 1 and 2).***

4. What about the discussion around culture, why not comparing indigenous people of low altitudes with those indigenous people at high altitudes, i.e., you can analyze proportions between low versus high, but among the same ethnic group, are there differences?

***Response: Thank you. We appreciate your comment and contribution to our study. We have analyzed the proportions of the outcome according to the ethnic groups. Please see line 232 and Supplementary Table 4.***
